# Real-World Outcomes of Antiangiogenic Therapy in Patients With Wet Age-Related Macular Degeneration

**DOI:** 10.7759/cureus.89937

**Published:** 2025-08-12

**Authors:** Diana Laura Solano Pineda, Italia Marín Hernández, Luis Gómez Morales, Adolfo Nuñez Cruz, Vanesa Flores Peredo, Erandi González Rubio Medina, Karen Medina Quero, Marco Antonio Vargas Hernández, Pedro Escalera Arroyo

**Affiliations:** 1 Immunology Laboratory, Military School of Graduate Studies in Health, Secretariat of National Defense (SEDENA), Mexico City, MEX; 2 Medicine, Faculty of Higher Studies Iztacala, National Autonomous University of Mexico, (UNAM), Mexico City, MEX; 3 Optometry, Military Hospital for Ophthalmologic Specialties, (SEDENA), Mexico City, MEX; 4 Optometry, Faculty of Higher Studies Iztacala, National Autonomous University of Mexico, (UNAM), Mexico City, MEX; 5 Research Department, Military School of Graduate Studies in Health, Secretariat of National Defense (SEDENA), Mexico City, MEX; 6 Retina Department, Hospital Regional Lic. Adolfo López Mateos, Institute for Social Security and Services for State Workers (ISSSTE), Mexico City, MEX; 7 Retina Department, Military Hospital for Ophthalmologic Specialties, Secretariat of National Defense (SEDENA), Mexico City, MEX

**Keywords:** age-related macular degeneration, anti-vegf, blindness, central macular thickness, oct, visual acuity

## Abstract

Introduction: Age-related macular degeneration (AMD) is one of the leading causes of irreversible blindness in developing countries. The treatment of choice is intravitreal antiangiogenic therapy. However, there are discrepancies between outcomes observed in real-world clinical practice and those reported in clinical trials such as RIVAL, MARINA, ANCHOR, and PrONTO.

Objective: The objective of this study was to explore real-world outcomes in visual acuity (VA) and central macular thickness (CMT) in patients with wet AMD treated with antiangiogenic therapy.

Methods: This was an ambispective, observational, and analytical study. A total of 110 patients (132 eyes) diagnosed with wet AMD were included. All had complete clinical records, optical coherence tomography reports, and VA data from January 2017 to June 2020.

Results: More than 80% of the eyes showed improvement in VA post-loading dose, along with a statistically significant reduction in CMT. The number of antiangiogenic injections ranged from 3 to 33, with an average follow-up of 20.8±12.5 months.

Conclusion: Treatment received in real-world clinical practice differs from the protocols established in multicenter trials, particularly regarding injection frequency. Despite these variations, a positive trend was observed in both visual improvement and CMT reduction at the end of follow-up.

## Introduction

Age-related macular degeneration (AMD) is the leading cause of visual impairment and blindness in industrialized countries [1,2]. The most severe visual loss is typically due to the neovascular (wet) form of AMD, which involves choroidal neovascularization (CNV) and associated macular edema [3]. This exudative variant is characterized by abnormal angiogenesis originating primarily from the choroid and less commonly from the choriocapillaris. The resulting abnormal vasculature is prone to leakage, leading to fluid accumulation, hemorrhage, and fibrosis, which can cause significant visual deterioration compared to the dry form of AMD [4]. The identification of vascular endothelial growth factor (VEGF) as a key molecule in the pathogenesis of neovascular AMD has led to the development of various therapies aimed at blocking this cytokine [5]. These treatments seek to restore retinal morphology and preserve or improve the function of the neurosensory retina in affected individuals. Initial treatment strategies included laser photocoagulation and photodynamic therapy (PDT) with verteporfin, both of which showed better outcomes than observation alone. However, laser therapy was associated with substantial vision loss, and PDT mainly stabilized vision rather than improving it [6]. The introduction of intravitreal anti-VEGF therapy revolutionized the management of patients with neovascular AMD and CNV. Currently, the most widely used anti-VEGF agents are ranibizumab and aflibercept [7]. Ranibizumab is a monoclonal antibody fragment (Fab) that binds to a specific isoform of VEGF, preventing receptor dimerization and subsequent neovascular processes. Aflibercept, on the other hand, functions as a soluble decoy receptor that binds VEGF-A and placental growth factor with greater affinity than their native receptors, thereby inhibiting their interaction and downstream activation [8].

Initial treatment protocols involved monthly intravitreal injections, as demonstrated in randomized clinical trials such as MARINA, ANCHOR, and VIEW [9]. Later regimens introduced a loading phase of three monthly injections followed by quarterly dosing, as seen in the PIER study. The PrONTO study implemented a loading schedule similar to previous trials but adopted a “pro re nata” (PRN) approach guided by optical coherence tomography (OCT) retreatment criteria. This regimen achieved comparable visual outcomes while requiring less than half the number of injections used in monthly protocols. The findings highlighted the value of OCT in guiding treatment decisions and demonstrated visual acuity gains consistent with those reported in phase III clinical trials.

In the treat-and-extend (T&E) regimen, anti-VEGF dosing intervals are extended based on individual responses in visual acuity and central macular thickness (CMT) post-loading phase [10]. Despite the demonstrated improvements in best-corrected visual acuity (BCVA) and reductions in CMT, real-world outcomes often differ from those reported in randomized controlled trials [11,12], underscoring discrepancies between clinical trial efficacy and everyday clinical effectiveness.

Objective

The objective of this study is to evaluate real-world outcomes in BCVA and CMT in patients with wet AMD (wAMD) treated with intravitreal anti-angiogenic therapy at the Retina Service of the Hospital Regional "Lic. Adolfo López Mateos," ISSSTE.

## Materials and methods

Study design

It is an ambispective, observational, and analytical study in patients with wAMD at the Adolfo López Mateos Regional Hospital, ISSSTE.

Ethical considerations

Prior authorization from the local Bioethics Committee (Protocol No. 257.2020) was obtained, and all procedures were conducted in accordance with the principles outlined in the Declaration of Helsinki. Informed consent was obtained from all participants and, when applicable, from their legally authorized representatives. Clinical records were reviewed from the archives of the Retina Service at Hospital Regional "Lic. Adolfo López Mateos," ISSSTE.

Sample size and selection

The sample size was estimated assuming a 90% expected anatomical response rate to anti-VEGF therapy in patients with neovascular AMD, with a 95% confidence level, a 5% margin of error, and a statistical power of 90%. Based on these parameters, a minimum of 138 participants was required. Although the final sample included 110 patients and 132 eyes, slightly below the calculated threshold, this difference is minimal and does not compromise the statistical validity or power of the study. Given the nature of the study, a non-probabilistic convenience sampling method was used, including all cases of wAMD treated with intravitreal anti-VEGF agents between January 2017 and June 2020.

A total of 214 patient records admitted to the retinal service were analyzed, of which 104 files were excluded. We included 110 patients (132 eyes) with a diagnosis of wAMD, treated with anti-VEGF intravitreal therapy ranibizumab 0.5 mg (Lucentis®), acquired from Novartis Mexico, and aflibercept 2 mg (Wetlia®), acquired from Bayer Mexico S.A. de C.V. These patients had complete clinical records, which included BCVA measured using standardized Snellen tables and CMT evaluated by OCT following the clinical protocols of the Retina Service.

Patients were excluded if they had a diagnosis other than wAMD, concomitant ocular pathologies, incomplete clinical records, known hypersensitivity or allergy to anti-VEGF injections, or discontinued follow-up or treatment.

BCVA and CMT assessment process

BCVA was assessed using Snellen charts under appropriate conditions to ensure accurate results. First, uncorrected visual acuity was measured for each eye individually. Then, objective refraction was performed with an autorefractometer. Subjective refraction was performed using a phoropter or trial lenses, adjusting spherical and cylindrical values based on the patient’s responses to find the best correction. Once the optimal correction was determined, BCVA was measured. The patient was asked to read the smallest line possible on the Snellen chart, and the last line with at least three out of five letters correctly identified was recorded. Visual acuity was expressed in fractional notation.

CMT was measured using spectral-domain OCT (Spectralis OCT; Heidelberg Engineering, Germany). This high-resolution imaging technique enables accurate retinal thickness quantification within the macular region, based on the subfields defined by the Early Treatment Diabetic Retinopathy Study (ETDRS) grid. The software automatically identified the internal limiting membrane and the retinal pigment epithelium to calculate the mean thickness in the central subfield. All values were expressed in micrometers (μm).

Statistical analysis

Descriptive statistics were performed, presenting the variables with measures of summary of central tendency and dispersion for quantitative variables, and absolute and relative frequencies for qualitative variables. No adjustments were made for confounding variables due to the descriptive nature of the study and the objective of knowing the real results according to the personalization of antiangiogenic treatment. The variables with normal distribution were presented in means and standard deviation (SD), while those that did not comply with normality were presented in median and interquartile range (IQR). Statistical normality tests were performed to determine the type of statistical analysis, since most of the study variables presented a non-normal distribution. Median and IQR were reported; however, mean and SD values were also demonstrated to facilitate comparisons with other studies. Contingency tables were used for qualitative variables and for those that were quantitative, such as visual acuity. For the comparison of CMT over time, Wilcoxon's paired signed range tests were used, and for visual acuity, Fisher's exact test, using the GraphPad Prism Software® statistical package version 8.02 (GraphPad Software, LLC, San Diego, California, USA) with a significance level of 0.05.

## Results

A total of 214 patient records from the Retina Service were reviewed, with 104 excluded due to: incomplete clinical records (missing initial or follow-up BCVA/OCT data), diagnoses other than wAMD, or additional comorbidities. The final analysis included 132 eyes from 110 patients with wAMD treated with intravitreal antiangiogenic therapy (ranibizumab and/or aflibercept) at Hospital Regional Lic. Adolfo López Mateos, ISSTE, between January 2017 and June 2020.

**Figure 1:**
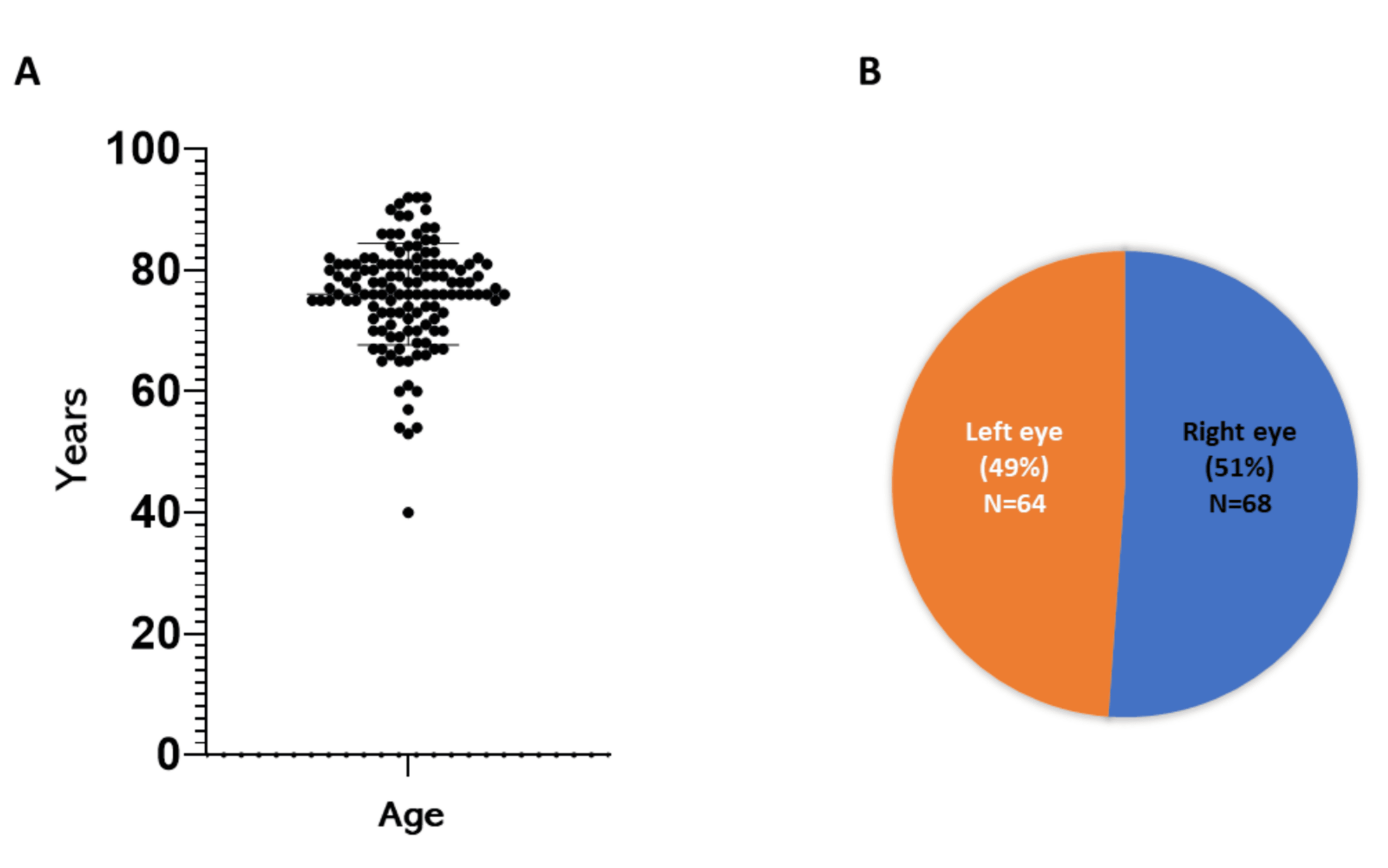
Characteristics of patients diagnosed with wet age-related macular degeneration (A) Age of patients diagnosed with wet age-related macular degeneration and treated with antiangiogenic therapy, with a higher concentration observed between 70 and 85 years of age. (B) Percentage of affected ocular side in eyes diagnosed with wet age-related macular degeneration.

The mean age of patients was 75.8±8.3 years (range: 53-92 years), with a predominance of female patients (n=61; 55.4%). Regarding laterality, 68 eyes (51%) had right-eye involvement and 64 eyes (49%) had left-eye involvement (Figures 1A, 1B).

Changes in visual acuity and CMT

Of the 132 eyes analyzed, 79 eyes (59.8%) were treated exclusively with ranibizumab (Lucentis®), comprising the largest treatment group. An additional 19 eyes (14.4%) were treated exclusively with aflibercept (Wetlia®). The remaining 34 eyes (25.8%) received combination therapy with both agents at different points during follow-up, depending on clinical evolution and OCT findings.

BCVA was evaluated using Snellen charts. Before treatment, 64.4% of cases had a BCVA of 20/400 or better. This percentage increased significantly to 80.3% post-loading dose (p=0.001) and further improved to 84.9% at the final follow-up visit (p=0.001, Table 1). Notably, no patients lost light perception during the course of treatment.

**Table 1:** Assessment of best-corrected visual acuity with a Snellen chart Percentage of patients assessed with Snellen charts to determine their best-corrected visual acuity (BCVA) at baseline, post-loading doses, and at the end of follow-up. Statistically significant improvement was observed at both time points (p=0.001). *A p-value of <0.05 was considered statistically significant. Fisher’s exact test was used for the analysis.

	Before the loading dose		After the loading dose		p*	Final follow-up visit		p*
BCVA (Snellen charts)	Number of patients (n)	%	Number of patients (n)	%	0.001	Number of patients (n)	%	0.001
<20/400	1	0.76	1	1.76		2	1.52	
20/40 to 20/60	8	6.06	13	9.85		18	13.64	
20/80 to 20/100	16	12.12	11	8.33		16	12.12	
20/140	13	9.85	18	13.64		26	19.7	
20/200	20	15.15	25	18.94		19	14.39	
20/400	27	20.45	38	28.79		31	23.48	
Count fingers	45	34.09	26	19.7		20	15.15	
Hand movement	1	0.76	0	0		0	0	
Perception of light	1	0.76	0	0		0	0	
No perception of light	0	0	0	0		0	0	

**Figure 2:**
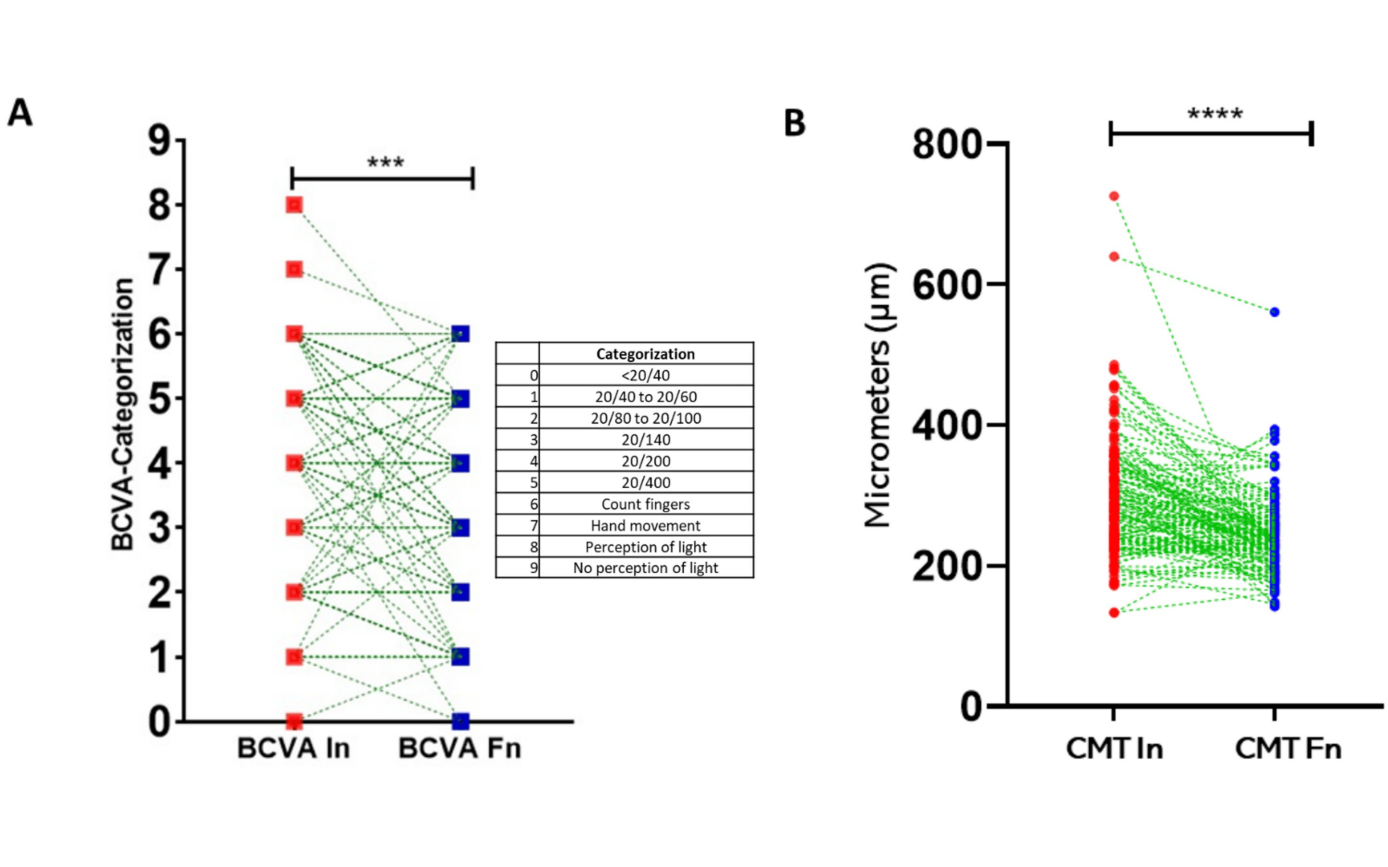
Changes in BCVA and CMT with the application of antiangiogenic therapy (A) Changes in BCVA at baseline and at the end of follow-up, with Snellen scale categorization and a significance level of p=0.0001. (B) Changes in CMT at baseline and at the end of follow-up, with a significance level of p=0.0001. ***p<0.001, ****p<0.0001 BCVA: Best-corrected visual acuity; CMT: Central macular thickness; In: Initial; Fn: Final

Treatment response was also assessed through changes in CMT, measured at three time points: baseline (before treatment), post-loading dose, and at the final follow-up visit (Table 2). The mean baseline CMT was 301.9±87.2 μm, which significantly decreased to 258±60.5 μm post-loading phase (p=0.001) and further declined to 240.5±60.5 μm at the last recorded visit (p=0.001, Figures 2A, 2B).

**Table 2:** Changes in central macular thickness over time Comparison of central macular thickness, as measured by optical coherence tomography, at baseline, post-loading dose, and at the end of follow-up with anti-angiogenic therapy. A statistically significant difference was observed (p=0.001). *A p-value of <0.05 was considered statistically significant. The Wilcoxon signed-rank test was used for the analysis. IQR: Interquartile range; SD: Standard deviation

	Before the loading dose		Post-loading dose		p*	Final follow-up visit	
	Mean ± SD	Median (IQR)	Mean ± SD	Median (IQR)		Mean ± SD	Median (IQR)
Central macular thickness (µm)	301.9±87.2	289 (243 to 343.5)	258±60.5	247 (223 to 287.5)	0.001	240.5±60.5	232 (201.5 to 258.5)

Anatomical outcomes and number of injections by treatment group

The mean follow-up period was 20.8±12.5 months (range: 1.4 to 42.7 months). Across all treatment groups, there was a significant and sustained reduction in CMT from baseline to the final visit, regardless of the specific treatment regimen applied (p=0.0001).

**Figure 3:**
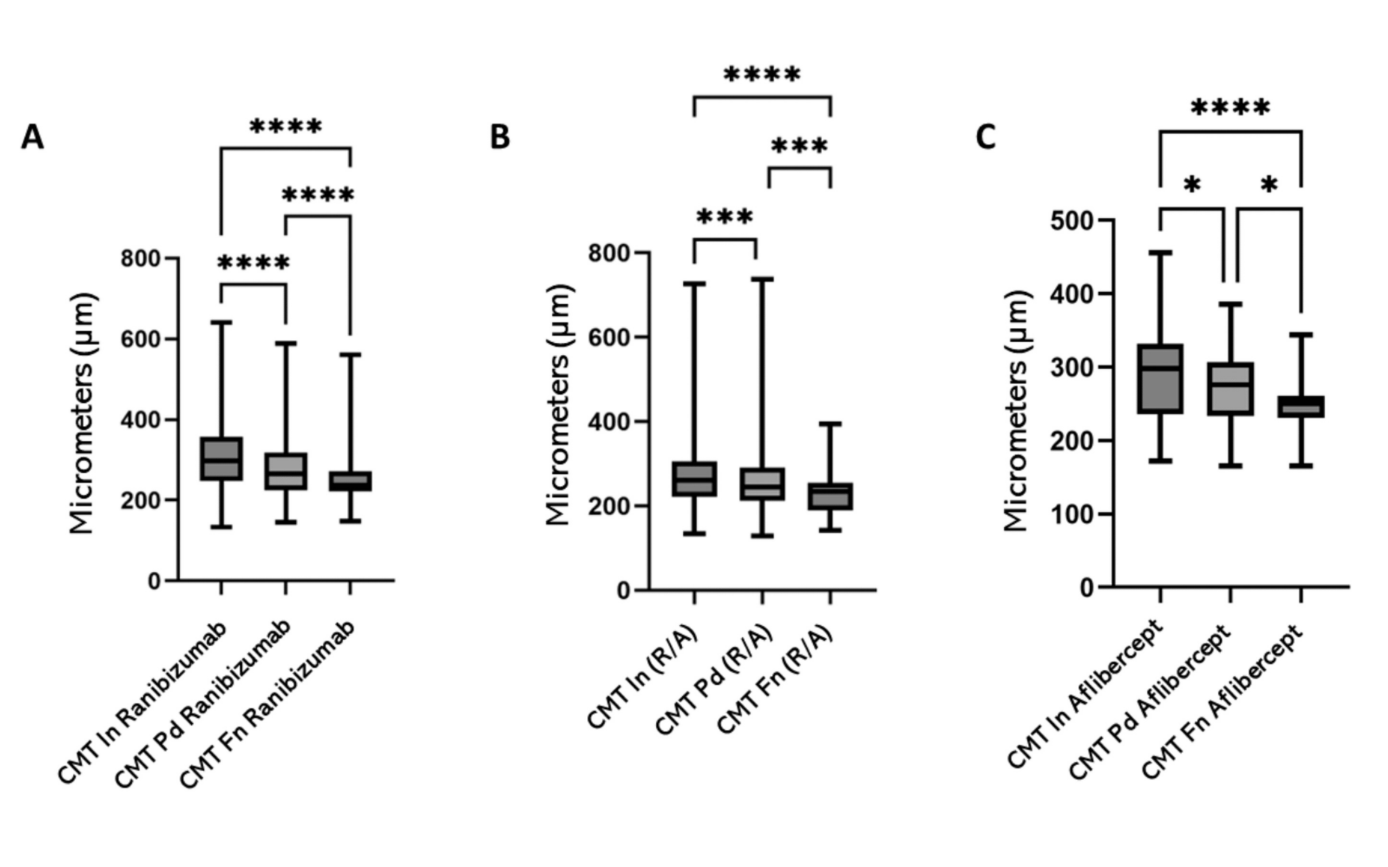
Changes in CMT with different antiangiogenics Changes in CMT at baseline, Pd, and Fn (end of the follow-up period) (A) with intravitreal injections of ranibizumab, (B) dual therapy (ranibizumab/aflibercept), and (C) aflibercept. *p<0.05, ***p < 0.001, ****p < 0.0001 CMT: Central macular thickness; Fn: Final; In: Initial; Pd: Post-load dose

In patients treated with ranibizumab, CMT decreased significantly from the post-loading phase to the final follow-up with a p=0.0001 (Figure 3A). A similar pattern was observed in patients who received combined therapy, with a significant reduction in CMT across all three time points: baseline, post-loading, and final visit (p=0.0001, Figure 3B). 

For patients treated exclusively with aflibercept, there was also a significant reduction in CMT between the initial and final measurements (p=0.0001); however, the reduction across all three time points was less pronounced, though still statistically significant with a p=0.0358 (Figure 3C).

**Figure 4:**
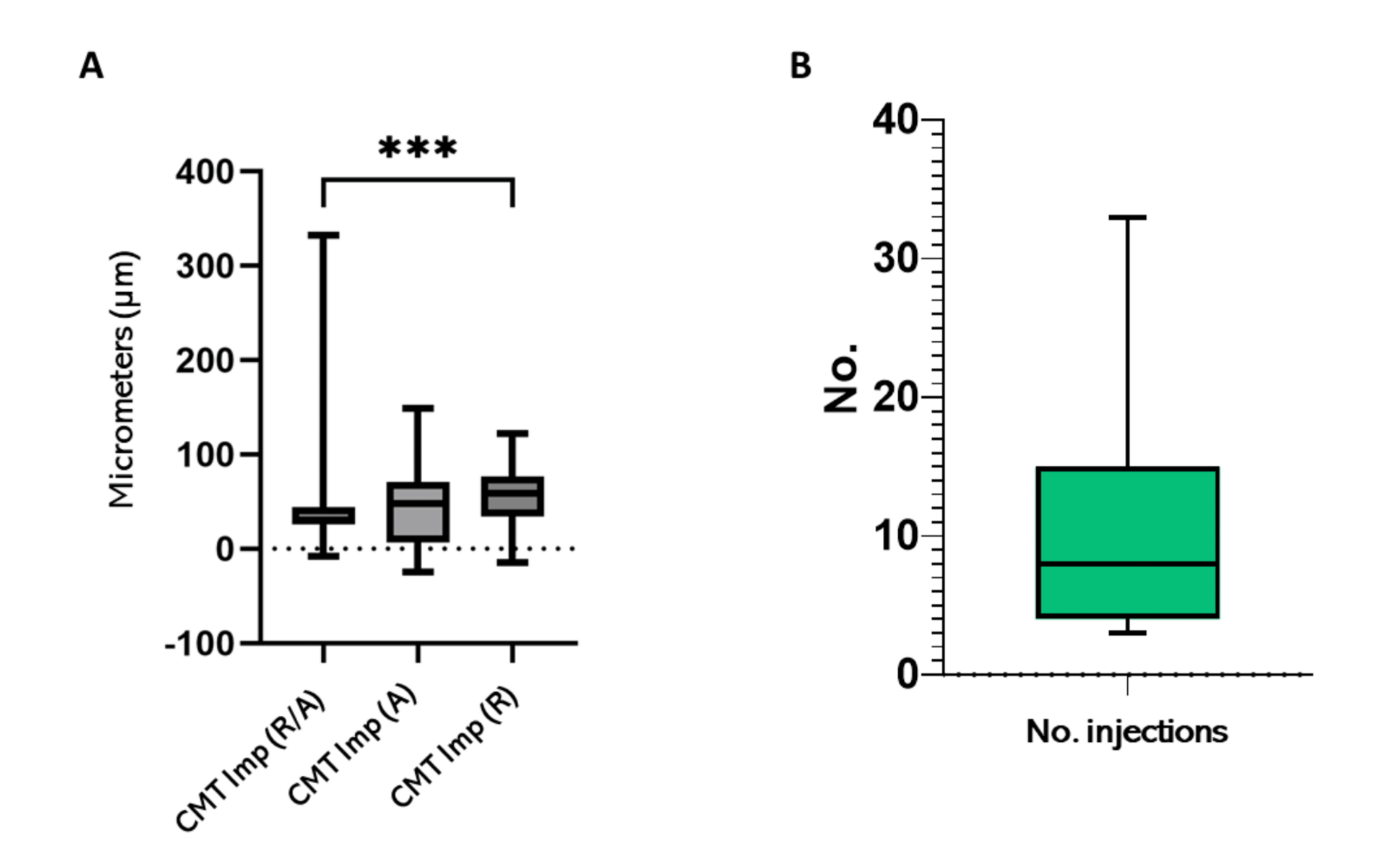
Number of injections and overall improvement in CMT (A) Final improvement in CMT of patients receiving different treatment regimens. CMT imp (R/A): ranibizumab/aflibercept, CMT imp A: aflibercept, and CMT imp R: ranibizumab. (B) Mean total number of injections (7.6±5.1) during follow-up with antiangiogenic therapy, ranging from 3 to 33 applications. ***p<0.001 CMT: Central macular thickness; Imp: Improvement; No. injections: Number of injections

Finally, the final improvement in CMT was evaluated and compared across the three different treatment regimens. Patients treated with ranibizumab and those who received combination therapy (ranibizumab/aflibercept) demonstrated a statistically significant improvement in CMT with a p<0.001. The average number of intravitreal injections administered was 7.6±5.1 (range: 3 to 33), as can be seen in Figures 4A, 4B.

## Discussion

This study demonstrated that patients with neovascular AMD (nAMD) treated with anti-angiogenic therapy (ranibizumab and aflibercept) experienced a reduction in CMT, accompanied by improvements in BCVA, following a “T&E” approach tailored to individual clinical and imaging-based needs.

More than 80% of eyes treated with anti-VEGF therapy showed significant visual improvement following the loading dose, defined as three monthly intravitreal injections; this improvement continued to increase by the final follow-up visit. These findings are consistent with previous evidence, such as the PrONTO study, which introduced the PRN regimen based on OCT-guided monitoring. This approach allowed for visual gains by anticipating CMT changes, thereby reducing the number of required injections while maintaining efficacy.

Regarding CMT, a statistically significant reduction was observed post-loading dose and at the end of the follow-up period. This anatomical stabilization supports the efficacy of intravitreal anti-VEGF injections. While BCVA is a key clinical marker of improvement, it is influenced by other factors, including retinal pigment epithelium tears, disease chronicity, conversion to dry AMD with geographic atrophy, and fibrosis, the last of which is potentially related to the total number of injections received.

In the aflibercept group, a significant reduction was observed between baseline CMT and final CMT. While the reduction in post-loading CMT was less pronounced, both post-treatment measurements showed statistically significant differences compared to baseline. This suggests an early and sustained therapeutic effect of aflibercept, supporting its anatomical efficacy under the T&E regimen tailored to individual needs. These findings are consistent with studies such as VIEW one and two, where aflibercept, administered every eight weeks following a loading phase, proved effective in maintaining CMT reduction [12].

In patients treated with ranibizumab or dual therapy (ranibizumab and aflibercept), CMT continued to decrease from the post-loading phase to the final follow-up. Anatomical improvement in those receiving combination therapy was significantly greater, suggesting that the use of two anti-VEGF agents may offer a more potent therapeutic effect. Our results regarding CMT reduction and visual acuity improvement are comparable to those described in monthly ranibizumab regimens in the VIEW and MARINA trials [13].

In relation to the number of anti-angiogenic injections administered, a variable range was observed, from 3 to 33 injections, with a mean of 7.6±5.1. This variability is likely due to differences in treatment regimens (PRN and T&E) or treatment interruptions due to patient or system-related factors. Despite differences in frequency and dosing intervals, the overall trend indicated both functional improvement (as measured by BCVA) and anatomical response (as assessed by CMT). In comparison, patients in the PrONTO study received 25 injections over 24 months, with a mean of 9.9 injections, while those in the MARINA and ANCHOR trials [13,14] received 24 injections over the same period. In contrast to our findings, Palazón-Cabanes et al. reported initial improvement in visual acuity at six months, followed by a gradual decline, which may be attributable to a lower number of injections (mean of 4.81 during the first year) [11].

The average follow-up period post-loading dose was 20.8±12.5 months (range: 1.4-42.7 months). The follow-up duration was calculated from the first anti-VEGF injection, representing a longer observation period with fewer injections compared to studies such as PrONTO, MARINA, and ANCHOR [10,13,14], which mandated strict monthly visits for two years.

In this study, BCVA was assessed using Snellen charts, which are commonly used in clinical practice in the Mexican population. However, this method differs from that employed in multicenter trials, where the ETDRS scale is preferred [15]. Furthermore, real-world treatment adherence in our cohort diverged from standardized trial protocols, often due to logistical or patient-specific factors. These limitations highlight the challenges of replicating rigid treatment regimens in everyday clinical practice.

Nonetheless, our findings support that even with variability in therapeutic adherence, patients achieved favorable clinical outcomes. These results underscore the adaptability and effectiveness of anti-VEGF therapies in real-world settings and reinforce their relevance for managing nAMD in diverse populations.

Limitations

Although the sample size in this study is adequate, future research would benefit from a larger, multicenter cohort to better represent the characteristics of patients with nAMD across different regions of the country. Additionally, the use of Snellen charts to assess BCVA presents a limitation when compared to the ETDRS scale, which is considered the gold standard. Adopting ETDRS methodology would provide more precise and comparable visual acuity measurements.

## Conclusions

This research study allows us to conclude that patients with wAMD experienced a statistically significant improvement in visual acuity in up to 80% of cases, which correlated with an improvement in final CMT in approximately 84% of treated eyes. The number of intravitreal injections administered varied depending on the individualized treatment plan, including differences in the drug used, frequency of administration, and interruptions due to various clinical or logistical factors. Despite not strictly adhering to the treatment intervals proposed by standardized regimens, none of the patients experienced a decline in visual acuity compared to their baseline. On the contrary, 80% showed visual improvement, and the remaining 20% achieved stabilization. Considering that the natural course of the disease leads to progressive vision loss, these outcomes are comparable to those reported in controlled multicenter trials and reflect the flexibility and efficacy of individualized anti-VEGF therapy in a real-world Mexican population. Long-term follow-up is recommended to monitor both visual acuity and macular thickness in these patients.

It is important to highlight that the value of treating nAMD extends beyond anatomical improvements; it significantly impacts patients' quality of life. Given that the natural outcome of this disease is progressive vision loss, preserving or improving vision directly affects the independence, functionality, and emotional well-being of those affected.
